# Therapies for the Treatment of Cardiovascular Disease Associated with Type 2 Diabetes and Dyslipidemia

**DOI:** 10.3390/ijms22020660

**Published:** 2021-01-11

**Authors:** María Aguilar-Ballester, Gema Hurtado-Genovés, Alida Taberner-Cortés, Andrea Herrero-Cervera, Sergio Martínez-Hervás, Herminia González-Navarro

**Affiliations:** 1 Health Research Institute Clinic Hospital of Valencia-INCLIVA, 46010 Valencia, Spain; abama4@alumni.uv.es (M.A.-B.); gehurge@alumni.uv.es (G.H.-G.); altacor@doctor.upv.es (A.T.-C.); anhecer@alumni.uv.es (A.H.-C.); 2 Endocrinology and Nutrition Service, Clinic Hospital of Valencia, 46010 Valencia, Spain; 3 Department of Medicine, University of Valencia, 46010 Valencia, Spain; 4 CIBERDEM (Diabetes and Associated Metabolic Diseases), 28029 Madrid, Spain

**Keywords:** cardiometabolic risk, incretin system, dipeptidyl peptidase 4, sodium-glucose-co-transporter 2 inhibitors, proprotein convertase subtilisin kexin 9

## Abstract

Cardiovascular disease (CVD) is the leading cause of death worldwide and is the clinical manifestation of the atherosclerosis. Elevated LDL-cholesterol levels are the first line of therapy but the increasing prevalence in type 2 diabetes mellitus (T2DM) has positioned the cardiometabolic risk as the most relevant parameter for treatment. Therefore, the control of this risk, characterized by dyslipidemia, hypertension, obesity, and insulin resistance, has become a major goal in many experimental and clinical studies in the context of CVD. In the present review, we summarized experimental studies and clinical trials of recent anti-diabetic and lipid-lowering therapies targeted to reduce CVD. Specifically, incretin-based therapies, sodium-glucose co-transporter 2 inhibitors, and proprotein convertase subtilisin kexin 9 inactivating therapies are described. Moreover, the novel molecular mechanisms explaining the CVD protection of the drugs reviewed here indicate major effects on vascular cells, inflammatory cells, and cardiomyocytes, beyond their expected anti-diabetic and lipid-lowering control. The revealed key mechanism is a prevention of acute cardiovascular events by restraining atherosclerosis at early stages, with decreased leukocyte adhesion, recruitment, and foam cell formation, and increased plaque stability and diminished necrotic core in advanced plaques. These emergent cardiometabolic therapies have a promising future to reduce CVD burden.

## 1. Introduction

Despite the existence of different cardiometabolic drugs, cardiovascular disease (CVD) remains the first cause of death worldwide [1]. A main classical risk factor is elevated blood levels of LDL-cholesterol (LDL-C) in the blood which are closely related to atherosclerosis [2], and constitute the first line of therapy [3]. However, the change in lifestyle patterns that promotes sedentarism and an aging of the population have raised the incidence of type 2 diabetes (T2DM), thus becoming a major emergent risk. T2DM features are frequently associated with hypercholesterolemia, dyslipidemia, hypertension, and obesity which altogether represent a cardiometabolic risk whose main complication is the atherosclerotic disease [4].

In recent decades, several mechanisms have been shown to contribute to aggravating atherosclerosis in T2DM patients [5]. Insulin resistance (IR) which plays a pivotal role in the onset of T2DM, increases endothelial cell (EC) dysfunction by diminishing the bioavailability of vasodilators like nitric oxide (NO) [6]. Other mechanisms include IR induced apoptosis in macrophages [4] and reduced survival in vascular smooth muscle cells (VSMC) [7] in lesions. Notably, these seem to be due to inflammatory signaling pathways that promote plaque instability and rupture [4,7,8]. Moreover, hyperglycemia contributes to glucotoxicity and exerts in the vascular bed a proatherogenic synergistic effect alongside dyslipidemia and hypertension. These and other mechanistic studies led to the hypothesis that anti-diabetic drugs might exert atheroprotection by acting directly in vascular cells, prompting many experimental studies and clinical trials to study the expanded use of these drug therapies in additional cardiovascular contexts [9,10].

**Figure 1:**
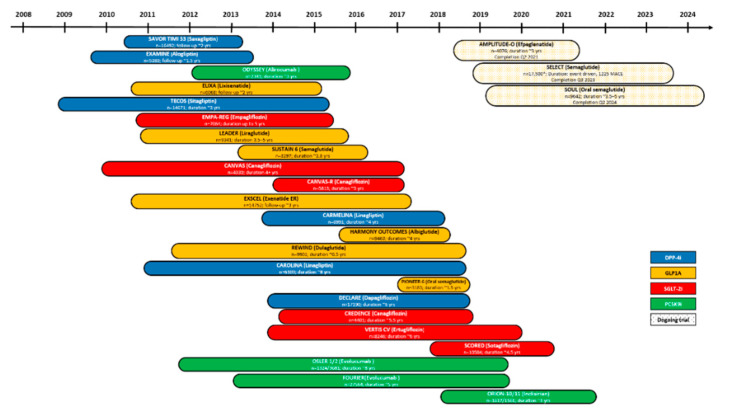
Clinical trials with cardiovascular end-points for T2DM patients in trials for incretin-based and SGLT2i therapies and with LDL-C percentage change from baseline end-points for trials testing PCSK9i. Based on https://clinicaltrials.gov/ct2/home (accessed 10 December 2020).

In the following sections, we will summarize the main current strategies for the management of carbohydrate and lipid metabolism and their relationship with CVD. These include incretin-based therapies, sodium-glucose co-transporter 2 inhibitors (SGLT2i), and proprotein convertase subtilisin kexin 9 (PCSK9) inhibitors. A schematic summary of clinical trials over the years is displayed in Figure 1.

## 2. Therapies Based on the Incretin System

### 2.1. Biology of the Incretins

Incretin hormones are small gut-derived peptides secreted by the endocrine cells of the intestine, mainly in the postprandial state, with potent insulinotropic actions. There are two main incretin hormones, the glucose-dependent insulinotropic polypeptide (GIP), secreted by the enteroendocrine K cells, and the glucagon-like peptide 1 (GLP1), secreted by the gut L cells of the distal small intestine and large bowel [11,12].

GLP1 is produced in response to nutrient intake, mainly sugars and fat, and its two active forms are the GLP1-(7–37) and the GLP1-(7–36)NH(2) generated by selective cleavage of the proglucagon molecule [13]. The GLP1 hormone exerts its effects through the GLP1 receptor (GLP1R) located in the gastrointestinal tract and pancreatic β-cells. However, it is also found in the heart, VSMCs, EC, immune cells, lung, kidney, and nervous system [13]. This protein is a G coupled receptor that mediates GLP1 actions to modulate glucose levels and body weight [13]. In β-cells GLP1 promotes the de novo synthesis and secretion of insulin by increasing cAMP and intracellular calcium levels. GLP1 also enhances β-cell number and mass through β-cell proliferation and neogenesis and decreases glucagon secretion in a glucose-dependent fashion that prevents hypoglycemia. These actions are concomitant to a reduction in food intake, gastric emptying, nutrient absorption and disposal, reduction of appetite by stimulation of the brain satiety center, and enhanced insulin sensitivity in periphery tissues [11]. GLP1 hormone is present at low concentrations in the circulation in fasting and inter-prandial state but, within minutes of food intake, its levels rise up to 2–3 times leading to postprandial insulin secretion [13]. GLP1 in the circulation has a very short half-life and, within 1.5 to 5 min, it is rapidly degraded by the dipeptidyl peptidase 4 (DPP4) into smaller peptides whose functions are not fully understood [14].

GIP production in the enteroendocrine K-cells of the proximal small intestine is mediated by the prohormone convertase (PC) one-third to a full-length peptide form. Alternatively, GIP can be processed by the PC2 to a C-terminal truncated form named GIP(1-30) [11] and it can be secreted under carbohydrate and fat regulation. In β-cells, GIP promotes insulin secretion by increasing cAMP and calcium intracellular levels and by regulating volatage-potassium Kv channel expression. Since its receptor, GIPR, is expressed in multiple tissues—such as the pancreas, gastrointestinal tract, adipose tissue, heart, testis, bone, lung, adrenal cortex, and central nervous system—other effects are under study. In the pancreas, GIP and GLP1 functions overlap, although inactivation of GIP in mice leads to impaired glucose tolerance and insulin release, hence indicating its metabolic relevance [11].

### 2.2. Degradation of the Incretins by Dipeptidyl Peptidase 4 (DPP4)

GLP1 and GIP incretins are cleaved by DPP4 during the passage across the hepatic bed. They are further processed in peripheral tissues into smaller metabolites to be finally eliminated by the kidney. DPP4, also known as CD26, is a transmembrane cell surface glycoprotein that cleaves N-terminal dipeptides position, mostly from GLP1 and GIP, thus holding an important antihyperglycemic effect. DPP4 is mainly expressed in exocrine glands and absorptive epithelium, hepatocytes, fibroblasts, leukocytes, and epithelial cells of the kidney and intestine [15]. It can be found membrane-bound or soluble. 

DPP4 is also an adipokine produced by proteolytic cleavage from fully differentiated adipocytes [16] in some settings. Its production correlates with the degree of IR, inflammation, adipocyte size, and the amount of visceral adipose tissue (VAT) [16,17]. Both insulin and TNFα increase up to 50% the release of soluble DPP4 and it is believed to participate in the crosstalk between adipocytes, macrophages, and the inflamed stroma-vascular fraction [16]. DPP4 levels increase with insulin and leptin and inversely correlate with age and adiponectin levels [16,18].

Consistently, rats with genetic DPP4 deficiency, such as the strain F344/DuCrj or Dark Agouti rats, display improved IR, glucose tolerance, lipid profile, and enhanced GLP1 activity. Likewise, DPP4-deficent mice have enhanced glucose tolerance, glucose-stimulated insulin, and GLP1 release, and improved insulin sensitivity and liver homeostasis [19]. Dendritic cells (DC) and macrophages from VAT, in mice and humans, exhibit elevated DPP4 levels which promote T cell activation, proliferation, and inflammation [20]. In ECs, DPP4 promotes T cell transendothelial migration [21] and in human VSMCs promotes inflammation. Hepatic DPP4 secretion is elevated in obesity and IR while specific hepatic DPP4 deletion through a short hairpin RNA (shRNA) strategy diminished adipose tissue macrophage infiltration and IR [22]. In agreement with these studies, T2DM patients also display reduced levels of GLP1 hormone and enhanced DPP4 in the circulation, and IR correlates with the levels of DPP4 released by adipose tissue depots [23].

Altogether indicates that incretin-based therapies display anti-inflammatory and cardiovascular benefits by mechanisms independently from their glucose-lowering effect. Soluble DDP4 has been shown to induce VSMC proliferation by activating mitogen-activated protein kinases (MAPK)-pathway and the nuclear factor kappa B (NF-κB)-mediated inflammation [24]. Likewise, GLP1 hormone activates thymocytes and T cell proliferation and maintains regulatory T cells [25]. Because of this, they have been tested as therapeutic drugs for the treatment of CVD in T2DM patients. Currently, there are two different strategies to increase the benefits of the incretin system: (1) inhibition of the peptidase DPP4 and (2) the use of GLP1 and GIP incretin analogues [26].

### 2.3. DPP4 Inhibitors as a Therapeutic Target for T2DM 

The gliptins are a family of DPP4 inhibitors developed as glucose-lowering agents that increase the half-life and bioavailability of the GLP1 incretin. These display fewer adverse side effects [27] and a low incidence of hypoglycemia [18]. The gliptins can be peptidomimetics and non-peptidomimetics, and both are extracellular competitive reversible inhibitors of the DPP4 substrate. These two groups differ in their half-lives, the strength of the effect, and therapeutic dose being the most currently prescribed: sitagliptin [28] saxagliptin [29], alogliptin [30], linagliptin [18,31], vildagliptin [32], anagliptin, and teneligliptin [18].

Most of them have shown a low frequency of hypoglycemia, pancreatitis, and pancreatic carcinoma, and no effects on heart failure (HF) rate or major adverse cardiovascular events (MACE) effects [27] except for alogliptin [30] and saxagliptin [29], which increase the rate of HF. 

### 2.4. Effects of DPP4 Inhibitors in CVD

Although the initial rational for DPP4 inhibition is the subsequent increase in GLP1 effects, other independent mechanisms have been reported. Thus, beneficial effects have been reported in adipose tissue, vascular ECs, and monocyte/macrophages during atherosclerosis and myocardial injury in preclinical studies as described below, some of them summarized in [33]. In Table 1, a description of the mechanisms found in the preclinical animal model studies and the effects on atherosclerosis are included for incretin-therapies. 

#### 2.4.1. Mechanisms of Gliptins in Experimental Atherosclerosis 

High levels of soluble DPP4 are found in atherosclerosis and many studies have shown that its inhibition results in restrained atherosclerosis progression (Table 1). 

Combination of DPP4 inhibitors with granulocyte-colony-stimulating factor (G-CSF) have shown protection against cardiovascular injury by increasing the number of endothelial stem cell and improving cardiac function [25]. The mechanism was an inhibition of DPP4-mediated degradation of the chemokine stromal cell-derived factor-1α (SDF-1α) which promotes endothelial progenitor cells (EPC) bone marrow mobilization to sites of cellular injury [55,56]. In this sense, SDF engineered to be DPP4-resistant showed improved blood flow in an animal model of peripheral artery disease [25].

In human umbilical vein endothelial cells (HUVECs), alogliptin induces endothelial nitric oxide synthase (eNOS), and AKT phosphorylation leading to enhanced NO production and improved homeostasis [24]. Likewise, alogliptin also promoted vascular relaxation via eNOS production of NO and endothelial-derived hyperpolarizing factor-mediated mechanisms [55]. Ex vivo DPP4 inhibition of human arteries from patients undergoing coronary artery bypass surgery reduced vascular oxidative stress and improved endothelial function [57]. DPP4 inhibitor treatment of HUVECs cultured under hypoxic condition prevented apoptosis in a CXCR4/STAT3-dependent manner [58].

In VSMCs, DPP4 activates MAPK and NF-κB increasing proliferation and production of iNOS proinflammatory cytokines [24]. Consistently, in *Apoe-/-* mice, anagliptin restrained atherosclerosis by suppressing VSMC proliferation [34].

DPP4 inhibition also modulates immune cells and inflammation. In diabetic patients, DPP4 inhibitors treatment resulted in a reduced production of reactive oxygen species and inflammatory mediators TNFα, JNK1, TLR2, TLR4, IL1β, and SOCS3 by monocytes [25]. DPP4 is highly expressed in bone marrow-derived CD11b+ cells. In *Lldr-/-* mice, DPP4 inhibitors downregulated proinflammatory genes and diminished aortic plaque macrophage content and lesion size [45]. In agreement with these results, treatment with linagliptin of high-fat diet-fed *Apoe-/-* mice, ameliorated atherosclerosis by inducing an anti-inflammatory phenotype in macrophages [35]. Likewise, anagliptin treatment restrained atherosclerosis by reducing macrophage plaque infiltration in cholesterol-fed rabbits [53] and suppressed inflammatory responses in macrophages in *Apoe-/-* mice [34].

In T cells, the non-cleaved membrane-bound DPP4 interacts with the T cell receptor (TCR)/CD3, promoting the phosphorylation cascade and antigen-presenting cell interactions engaging NF-κB inflammatory pathway activation [17]. 

#### 2.4.2. Clinical Studies on DPP4 Inhibitors in T2DM with CVD

Both sitagliptin and anagliptin are being evaluated in clinical trials for their potential in the regulation of lipid metabolism and CVD in T2DM patients (Table 2). Anagliptin has been shown to decrease LDL-C triglycerides, total cholesterol, and non-HDL-C levels in a mechanism independent from its hypoglycemic effects [59]. Other gliptins increase adiponectin levels and reduce intestinal cholesterol absorption [18]. 

Over the years, the cardiosafety of DPP4 inhibitors in T2DM patients with high cardiovascular risk has been confirmed in several clinical trials (Table 2). The SAVOR-TIMI 53 trial [29], the EXAMINE trial [30], the TECOS trial [60], and the CAROLINA [61] and CARMELINA [31,62] trials, that evaluated saxagliptin, alogliptin, sitagliptin, and linagliptin treatment, respectively, are considered the most important. Nevertheless, no cardiovascular benefits were reported in comparison with the placebo group. In fact, in the SAVOR-TIMI 53 trial, an increase in the rate of HF in patients with no previous history, was detected in the treated groups. The SAVOR-TIMI 53 trial [29], the EXAMINE trial [30] and TECOS trial [60] showed very low frequencies of hypoglycemia, pancreatitis, and pancreatic carcinoma [27]. However, the CARMELINA trial did not detect any effect on kidney disease or cardiovascular and kidney events.

### 2.5. GLP1 Analogues as Therapeutic Strategies

#### 2.5.1. Development of Drugs Based on GLP1 and Rational Design 

Given that GLP1 stimulates insulin secretion, soon after its discovery, this incretin became useful for T2DM treatment [79]. The native peptide has no clinical applicability due to its short plasma life-time of less than 2 min [80]. Therefore, the GLP1 analogues (GLP1A) approved for clinical use [81] are either derivatives of the human native GLP1, with chemical modifications to increase its stability and half-life [82] or peptides based on exendin-4. Exendin-4 is a GLP1A peptide isolated from the saliva of the lizard *Heloderma suspectum* [83], whose an amino acidic identity of 53% to the mammalian GLP1 that allows it to bind to the human GLP1 receptor [11]. 

Exenatide was the first GLP1A approved in 2005 and it is a synthetic version of the exendin-4. It has a half-life of up to 2.4 h, 10 times longer than endogenous GLP1, because its resistance to human DPP4 degradation [81,82]. Since the first generated GLP1As have a subcutaneous administration twice daily, other compounds have been developed to extend durability [84]. The optimization approach was addressed to keep the human GLP1 backbone to avoid immunogenic problems [84] and to get DPP4 action resistance through GLP1-(7-36) region modifications [26,81]. These modifications consist in the replacement of the penultimate alanine at the N-terminal end of the peptide by a glycine, serine, D-alanine, or by the most optimal chemical group, aminoisobutyric acid (Aib) [80], since it does not interfere with GLP1 receptor binding [85]. Other modifications were a replacement of the histidine residue at the N-terminal end by a glucitol group or by performing a deamination [80]. To increase the GLP1 half-life, the addition of fatty acids to the C-terminal domain was also performed to allow binding to the albumin and protection to renal filtration [26,86].

By combining all of the above-mentioned modifications, the approved GLP1As were designed and generated progressively over time: liraglutide (2009), exenatide (2005) and exenatide once-weekly (2011), lixisenatide (2013), albiglutide (2014) dulaglutide (2014), and semaglutide (2017), whose specific modifications can be found elsewhere [79,81].

These compounds have shown favorable effects in experimental and clinical studies with the limitation of parenteral administration [26,82]. Therefore, current research is focused on developing novel small GLP1As to be administered orally [26,84]. Among these, oral semaglutide stands out, which is a small acetylated peptide [79] whose oral daily administration has recently been approved in combination with sodium *N*-(8-(2-hydroxybenzoyl) amino) caprylate (SNAC) [84].

#### 2.5.2. Studies in Preclinical Models of T2DM, Atherosclerosis, and CVD 

As summarized in Table 1, studies in rodents and rabbits have shown beneficial actions of GLP1As in atherosclerosis, vascular injury, and cardiac function. Liraglutide treatment in diabetic rats led to diminished atherosclerotic lesion formation and intima-media thickening through a decrease of macrophage secretion of ER-stress induced microvesicles [51]. Similarly, the expression of GLP1 by adenovirus-mediated delivery in diabetic rats reduced intima-media thickening, VSMC and monocyte migration, and inflammatory processes [52]. 

In *Apoe-/-* mice, treatment with both native GIP and GLP1 incretins diminished atherosclerosis by suppressing macrophage foam cell formation [37]. In *Apoe-/-* mice liraglutide suppressed acyl-coenzyme *A cholesterol acyltransferase* 1 (*Acat*1) expression, foam cell formation and decreased atherosclerosis [38], and inhibited progression of early-onset atherosclerosis lesions in a GLP1R-dependent fashion [87]. Liraglutide administration also improved cardiac function through reduced cardiac fibrosis, hypertrophy, and necrosis by ameliorating endothelial NOS expression and ER-stress response in 45% high-fat diet (HFD) fed C57Bl6 mice [47]. 

Studies conducted by us have shown diminished atherosclerosis by lixisenatide and liraglutide in a mouse model of atherosclerosis and IR, the *Apoe-/-Irs2+/-* mice. Moreover, lixisenatide generated more stable plaques with thicker fibrous caps, smaller necrotic cores, and reduced inflammatory cell infiltration [43]. Lixisenatide also promoted macrophage polarization toward a pro-resolving phenotype in a STAT3-dependent manner [43]. A similar mechanism was found in *Apoe-/-* mice where liraglutide modulated polarization of macrophages toward a pro-resolving phenotype too [39]. Likewise, exenatide exerted protective effects in cultured macrophages via STAT3 activation which promoted adiponectin secretion when co-cultured with 3T3-L1 adipocytes [88]. In another investigation, liraglutide, and semaglutide treatments reduced lesion development in both *Apoe-/-* and *Low density lipoprotein-deficient* (*Ldlr-/-*) mice fed western diet, through major changes in inflammatory markers in aortic tissue [44]. Similarly, exenatide beneficial effects in atherosclerosis in *Apoe-/-* mice were attributed to reduced plaque oxidative stress, inflammation, and proteolysis in mice under chronic stress [40]. In the same line of results, lixisenatide treatment of Watanabe heritable hyperlipidemic (WHHL) rabbits prevented plaque growth and instability by decreasing macrophage infiltration, calcium deposition, and necrosis, as well as increased fibrotic content in VSMC-rich plaques [54]. On the other hand, exendin-4 treatment also decreased lesion size in *Apoe-/-* mice through a reduction of monocyte adhesion and expression of pro-inflammatory cytokines in activated macrophages via cAMP/PKA pathway [41]. Exendin-4 also diminished liver inflammation and atherosclerotic lesions through monocyte/macrophage recruitment and adhesion, and foam cell formation in the atherosclerotic mouse model APOE*3-Leiden.CETP [42].

In in vitro studies, treatment of HUVECs with exendin-4 [89] or liraglutide [90] incremented the activity, phosphorylation, and protein levels of eNOS, thus protecting against proatherosclerotic factors [89,90]. Besides, liraglutide in HUVECs diminished high-glucose-induced NF-kB phosphorylation and associated endothelial dysfunction [90]. Liraglutide also alleviated TNFα and LPS-mediated monocyte adhesion in cultured human aortic endothelial cells (HAECs) by increasing Ca^2+^ and CAMKKβ/AMPK activities and diminishing E-selectin and VCAM1 expression [91]. On the other hand, exendin-4 reversed glucolipotoxic gene dysregulation in diabetic human coronary artery endothelial cells (HCAECs) by upregulating eNOS and proangiogenic genes, while downregulating inflammatory, prothrombotic, and apoptosis genes [92].

In ischemia-reperfusion injury rat models (Table 1), prolonged treatment with lixisenatide reduced infarct-size and improved cardiac function [49]. Furthermore, in restenosis mouse and rat models, exendin-4 and lixisenatide reduced VSMC proliferation and neointimal hyperplasia, respectively [50]. In a mouse model of myocardial injury, liraglutide pretreatment reduced mortality and infarct size by enhancing cardioprotective genes such as PPARβ-δ, Nrf-2, and HO-1. Moreover, liraglutide-treated cardiomyocytes displayed diminished caspase-3 activation and increased cAMP production [48]. In another study, which employed an arterial hypertension angiotensin II-mouse model, liraglutide reduced leukocyte rolling and infiltration of myeloid neutrophils into the vascular wall [46].

#### 2.5.3. Clinical Studies on GLP1-Based Strategies in T2DM with CVD 

Many clinical trials based on GLP1 analogues have been designed to approve their use in CVD complications in T2DM subjects (Table 2). 

In the ELIXA (Evaluation of Lixisenatide in Acute Coronary Syndrome [NCT01147250]) trial, T2DM patients with a recent acute coronary syndrome were enrolled to test a daily injection of lixisenatide, an exendin-4-based analogue, for 25 months. Results showed no effects on MACE or other serious adverse events in T2DM patients who had recently suffered an acute coronary event [63]. The HF hospitalization rates and death remained unchanged but systolic blood pressure (SBP) and heart rate were reduced [63].

The effect on cardiovascular events of weekly injections of exenatide, another exendin-4 based analogue, was evaluated in the EXSCEL [NCT01144338] clinical trial. The study included T2DM patients of which 70% exhibited CVD and 30% did not displayed any CVD. In this study CVD was defined as atherosclerosis peripheral artery disease, coronary artery disease or stroke. The follow-up of patients, which were randomly treated with once-weekly exenatide (2 mg) or placebo for 5 years, did not show differences in MACE. A modest decrease in SBP and LDL-C was seen but treated patients also showed enhanced heart rate. The lack of effect was related to a shorter follow-up period and lower glycated hemoglobin baseline levels in the patients that in the other trials [64]. Once-weekly exenatide did not affect retinopathy or renal outcomes and produced a modest reduction in CV risk in subjects with mild loss of kidney function (estimated glomerular filtration rate, eGFR ≥ 60 mL/min/1.73 m^2^) [65].

In the LEADER (Liraglutide Effect and Action in Diabetes: Evaluation of Cardiovascular Outcome Results [NCT01179048]) clinical trial, the human GLP1-based analogue liraglutide or placebo were administered once daily as a subcutaneous injections to T2DM patients at high cardiovascular risk with a median follow-up of 3.8 years. In the first analysis, the treated group had lower rates of cardiovascular events and death from any cause but they also suffered more episodes of gallstone disease. The liraglutide group displayed a reduced risk of MACE, defined as cardiovascular death, non-fatal stroke, and non-fatal, first or recurrent MI. Analysis of vascular function showed decreased SBP and microvascular retinal and renal complications but also augmented DBP and heart rate [66]. A second analysis of this trial indicated the same benefits for polyvascular and single vascular disease in T2DM patients, independently of a previous history of MI [67]. In a posthoc analysis which took into account T2DM patients with high cardiovascular risk—due to MI, stroke, or atherosclerosis—liraglutide reduced cariovascular outcomes [68]. In another analysis of the LEADER trial, there was no evidence of decreased cardiovascular outcomes in patients with MI treated with liraglutide [69]. In a third clinical study, liraglutide treatment in T2DM patients resulted in a lower rate of diabetic kidney disease development and progression than placebo [70].

Likewise, semaglutide, whose structure is also based on human GLP1, injected once-weekly at two doses (0.5 mg or 1.0 mg) for 104 weeks in the SUSTAIN-6 trial (NCT01720446) reduced the rate of cardiovascular death and non-fatal MI and stroke in patients with T2DM at high cardiovascular risk. Consistent with the other GLP1A, SBP was reduced but mean pulse rate enhanced [71]. However, in the PIONEER 6 (NCT02692716) trial, once-daily oral semaglutide did not alter the cardiovascular risk profile of T2DM patients at high cardiovascular risk, but decreaed SBP and LDL-C parameters. Moreover, gastrointestinal adverse events were more common in patients receiving oral semaglutide than patients with placebo [72].

Another chemical variant of human GLP1, albiglutide, which consists of two tandem copies of modified human GLP1 bound to human albumin, showed increased effectivity due to its long-acting effect. In the Harmony Outcomes (NCT02465515) clinical trial, albiglutide in subjects of 40 or more years of age with T2DM and CVD, showed reduced MACE, SBP, and improved glomerular filtration rate in a follow up of 1.6 years. However, no effect was observed in death from cardiovascular causes [73]. 

A long-acting GLP1A, called dulaglutide, has also been generated. This analogue is the human GLP1 peptide covalently linked to a Fc fragment of a human IgG4 that protects against DPP4 degradation. The REWIND (NCT01394952) clinical trial, which tested weekly subcutaneous injection of dulaglutide in T2DM patients with a previous cardiovascular event or cardiovascular risk factors, showed no effect in all-cause mortality rate but reduced the risk of CV outcomes, SBP, pulse and arterial pressure although increased heart rate [74]. A subsequent analysis showed that weekly subcutaneous dulaglutide injections reduced total cardiovascular or fatal event burden [75].

Ongoing clinical trials are the SURPASS-CVOT (NCT04255433) with an estimated enrollment of 12,500 participants and the AMPLITUDE-O (NCT03496298) with 4076 patients. In the first trial, the effect in MACE of two incretins, tirzepatide (LY3298176) and dulaglutide is being compared in T2DM patients and the completion date of the study is in October 2024 (https://clinicaltrials.gov/NCT04255433). The second trial studies the effect of efpeglenatide in T2DM patients at high CVD risk and its estimated completion date is April 2021 (https://clinicaltrials.gov/NCT03496298).

### 2.6. GIP1 Emergent Therapies

#### 2.6.1. Development of Drugs Based on GIP and Rational Design

Currently, there are no clinical therapies using GIPR agonists [93]. Experimental results of GLP1R/GIPR dual co-agonists derived from intermixed incretin sequence have shown augmented antihyperglycemic and insulinotropic effect compared with specific GLP1 agonist in *db/db* mice, ZDF diabetic rats, monkeys and humans [94,95]. Moreover, these dual agonists have been shown to reduce plasma biomarkers of cardiovascular risk in diet-induced obese mice [95,96]. 

#### 2.6.2. Investigations of GIP Therapies in Preclinical Models 

GIP has been related to different signalling pathways in vascular cells with both anti-atherogenic via NO production, or pro-atherogenic effects through increased endothelin-1 and VSMCs osteopontin expression [93]. In HUVECs cultures GIP/GIPR and GLP1/GLP1R interactions reduce the advanced glycation end products (AGEs) receptor (RAGE) expression, blocking the signalling pathways associated with diabetes-associated vascular damage [97]. 

On the other hand, as shown in Table 1, GIPR levels are diminished in diabetic experimental models and consequently, administration of GIP in *Apoe-/-* and *db/db* diabetic mice reduced atherosclerotic plaque lesions and foam cell formation [37,98]. Furthermore, the administration of GIP active forms to *Apoe-/-* mice increased atherosclerotic collagen content [36], suppressed VSMCs proliferation and reduced aortic endothelial expression of MCP1, VCAM1, ICAM1, and PAI1 [37,93] and monocyte migration and macrophage activation in a NFkB-dependent manner [36]. Other observed actions are the blocking of proinflammatory monocyte aortic infiltration, the decrease in *Cd36* and *Acat1* expression, foam cell formation, LPS-induced IL-6 secretion, and MMP-9 activity [36,37,93]. 

#### 2.6.3. Studies in Humans with T2DM and CVD

GIP serum levels are elevated in patients with atherosclerotic vascular disease [36]. In humans, physiological doses of GIP increased heart rate, arterial blood pressure, and blood levels of osteopontin CCL8 and CCL2, accordingly with reduced CCL2/CCR2-mediated migration of cultured human monocytes (THP-1 cells) [93]. 

GLP1R/GIPR co-agonist NNC0090-2746 tested in T2DM patients in a randomized, placebo-controlled, double-blind phase 2 trial, administered subcutaneously once a day, showed an important effect in glycemia control and diminished body weight, cholesterol levels, and leptin [76] (Table 1). On the other hand, the dual co-agonist LY3298176 (tirzepatide) biased towards GIPR agonism obtained promising results as antidiabetic treatments in T2DM clinical trials as it showed improvements in glycemic and body weight control although is also showed an increased pulse rate [77,78,96]. Further research is needed about their cardioprotective effects, while interest in new GLP1R/GIPR co-agonists development increases [94].

## 3. Therapies to Inhibit Sodium-Glucose Co-Transporter 2 

### 3.1. Structure of Sodium-Glucose Co-Transporters and Mechanism of Action

Human sodium-glucose co-transporters (SGLTs), encoded by *SLC5*, are a family of 12 secondary active glucose transporters, being the SGLT1 and SGLT2 the most important. The core domain structure of this LeuT-superfamily is a five-helix inverted repeat motif [99,100] with a substrate-binding site in the middle of the protein and with an outer and an inner gate. Glucose adsorption by SGLT1 mostly takes place in the intestine and SGLT2 plays a key role in the renal glucose reabsorption with a highly similar sodium-coupled sugar transport mechanism.

SGLTs transport glucose across the brush-border membrane of the proximal renal tubule segments. SGLT2 mostly located in the S1 segment accounts for up to 90% of glucose reabsorption and SGLT1 in the S3 segment accounts for 10% of reabsorption [100,101]. The transport mechanism consists of Na+ binding to the transporter in the apical side of the epithelial cells, the opening of the external gate and glucose binding. This is followed by the closing of the external gate, the opening of the inner gate, the release of both substrates to the cytosolic side of the cell and the adoption of the ligand-free conformation [101,102]. Through the basolateral membrane of the epithelial cells, GLUT2 transporters and Na+/K+ pump alleviate the intracellular accumulation of sodium and glucose by delivering both molecules in the blood.

### 3.2. Development of SGLT Inhibitors

The rational for developing SGLT2 inhibitors (SGLT2i) is the existence of up to 50 human mutations of SGLT2 resulting in renal glucosuria and important urinary glucose losses. These suggested that SGLT2i could be of use for hyperglycemic states such as T2DM patients [103]. 

Phlorizin, a glucoside of phloretin found in the apple tree, was the lead of the SGLT inhibitors as studies on IR diabetic rats showed that its administration subcutaneously normalized plasma glucose profiles and insulin sensitivity [104]. Because of its poor solubility, low bioavailability, and unselective SGLT1/2 inhibition, it was set aside as a therapeutic candidate. Their synthetic derivates, T-1095A and T-1095, had better results though their clinical development did not continue because of the same limitations [103,105].

Novel molecules with a C-glycosylation modification that conferred the phlorizin resistance to hydrolysis by endogenous β-glucosidases, increasing their half-life, were developed [103]. Selective SGLT2i based on this meta-C-glycosylated diarylmethane pharmacophore led to dapagliflozin, canagliflozin, empagliflozin, and ertugliflozin. The gliflozins have a higher selectivity profiles for SGLT2 over SGLT1, except for sotagliflozin with a selectivity for SGLT1 of about 20:1. Sergliflozin is another SGLT2i based on benzylphenol glucoside that did not undergo further development after phase II (https://adisinsight.springer.com/search). Others SGLT2i are ipragliflozin, tofogliflozin, and luseogliflozin, which are approved and available in Japan [106].

Effects of gliflozins also include actions on pancreatic islet cells and modulation of endogenous glucose production in insulin-sensitive tissues. Hence, empagliflozin treatment of T2DM subjects improved β-cell function [107], while dapagliflozin affects glucagon secretion by α-cells [108]. Moreover, T2DM patients treated with dapagliflozin and empagliflozin have shown an endogenous production of glucose and improved insulin sensitivity [107,109].

### 3.3. Effect of Gliflozins in Preclinical Models of CVD

Tahara et al. [110] studied different gliflozins (dapagliflozin, empagliflozin, canagliflozin, luseogliflozin, ipragliflozin, tofogliflozin) in KK/Ay T2DM mice (Table 3). The treatment with these gliflozins decreased hyperglycemia, lowered plasma levels of inflammatory mediators and improved endothelial dysfunction associated with human atherosclerosis.

Studies with empagliflozin in diabetic ZDF rats and *db/db* mice also showed an improvement of vascular stiffness and endothelial function by preventing oxidative stress, AGE-dependent signaling and inflammation [111,112]. Likewise, dapagliflozin treatment of C57BLKS/J-lepr*db*/lepr*db* mice, alleviated arterial stiffness and endothelial and VSMC dysfunction. Interestingly, a decrease in circulating markers of inflammation and alterations in microbial richness and diversity were reported [113].

In vascular disease, dapagliflozin decreased atherosclerosis in streptozotozin-induced diabetic *Apoe-/-* mice, but not in non-diabetic *Apoe-/-* mouse counterparts. Moreover, analysis of peritoneal macrophages from dapagliflozin-treated diabetic *Apoe-/-* mice and ipragliflozin-treated *db/db* mice showed decreased cholesterol-ester accumulation, suggesting less macrophage foam cell formation capacity compared. Both SGLT2i normalized the expression of *lectin-like ox-LDL receptor1* (*Lox1*), *Acat1*, and *ATP-binding cassette transporter A1* (*Abca1*) in peritoneal macrophages from diabetic *Apoe-/-* and *db/db* mice. These results suggested that SGLT2i exert an anti-atherogenic effect in diabetic conditions by modulating genes involved in cholesterol accumulation in macrophages [114].

Studies carried out in *Apoe-/-* mice showed that dapagliflozin improved EC function without variations in plasma glucose concentrations [115] and that empagliflozin reduced plaque formation in the aortic arch and ameliorated insulin sensitivity [116]. A decrease in inflammatory mediators, like VCAM and NFκB [115] and TNFα and IL6 [116] were also observed in these studies. These results point to an atheroprotection through anti-inflammatory mechanisms. Consistently, high-fat diet fed *Apoe-/-* mice, treated with canagliflozin, developed smaller and more stable plaques and displayed decreased *Vcam* and *Mcp1* expression [117].

In the T2DM mouse model, the *ob/ob* mice, dapagliflozin improved left ventricular function, attenuated activation of the Nlrp3 inflammasome and reduced inflammatory mediators by an AMPK-dependent mechanism. These changes were observed also in cardiomyofibroblasts derived from mice and were independent of the glucose-lowering effect [118]. 

### 3.4. Clinical Studies of Gliflozins in HF and CVD

In the EMPA-REG OUTCOME (NCT01131676) study, the empagliflozin treatment of patients with T2DM and CVD reduced MACE, death and hospitalization for HF. Both SBP and DBP were reduced while HDL-C and LDL-C levels were increased. However, it did not ameliorate non-fatal MI and stroke, and patients showed an increased rate of genital infection [119] (Table 3).

In the CANVAS (NCT01032629) and CANVAS-R (NCT01989754) program, canagliflozin treatment of patients with T2DM at high CV risk resulted in diminished MACE but it did not alter the occurrence of CV death or overall mortality. Like empaglifliozin, it enhanced the levels of HDL-C and LDL-C and the treated patients displayed a greater risk of fractures and amputations [120].

Differences between EMPA-REG OUTCOME and CANVAS trial might be due to the study design. Thus, in the EMPA-REG all subjects had prior CV disease and additional treatments (i.e., statins, RAS inhibitors, and acetylsalicylic acid), while in the CANVAS study only 65% of patients had prior CV disease [127].

Canagliflozin tested in T2DM and albuminuric chronic kidney disease patients in the CREDENCE (NCT02065791) clinical trial, demonstrated that, besides a lower risk of cardiovascular death, MI or stroke, this drug also diminishes the risk of end-stage kidney disease without changes in the amputation and fracture rates [121].

In the DECLARE-TIMI 58 (NCT01730534) trial with T2DM and CVD patients, dapagliflozin treatment failed to reduce MACE, although the rates of cardiovascular death or hospitalization for HF were lower. In this study reductions in SBP and DBP were described for dapagliflozin-treated patients [122]. Dapagliflozin in the DEFINE-HF (NCT02653482) trial with patients displaying chronic HF, both patients with or without T2DM, did not reduce HF determined as B-type natriuretic peptide levels [123].

Similar to dapagliflozin in the DECLARE-TIMI 58, ertugliflozin in the VERTIS CV (NCT01986881) trial, which included T2DM and CVD patients, did not reduce MACE or neither modify the death rate attributed to CV causes. However, ertugliflozin reduced SBP [124].

Two trials were carried out with sotagliflozin, the SCORED (NCT03315143) clinical trial [125] with TD2M chronic kidney disease and CVD participants, and the SOLOIST-WHF (NCT03521934) trial [126], with TD2M patients with HF. In both trials, a decrease in MACE was observed, but patients in the treatment group of the SCORED trial suffered more genital infections and volume depletion, in addition to diarrhea and hypoglycemia in both trials. 

## 4. Lipid-Lowering Therapies Based on the Proprotein Convertase Subtilisin Kexin 9 Inhibition

As mentioned in the introduction one main mechanism for the regulation of blood LDL-C levels relies on its clearance by LDLR located on the surface of hepatic cells. This receptor binds the apoprotein B-100 (Apo B-100) present in LDL, VLDL, and IDL particles. After LDL binding, the receptor leads to the clathrin-mediated endocytosis of the ligand-receptor complex, which finishes in LDL degradation into amino acids and cholesterol. LDLR undergoes internalization and degradation by the proprotein convertase subtilisin kexin 9 (PCSK9), thus playing a pivotal role in the regulation of lipoprotein clearance [128]. *PCSK9* gene was identified in 2003 in a family with Familial hypercholesterolemia (FH), an autosomal dominant disease with elevated LDL-C in blood [129]. FH was attributed to two missense mutations in the coding region of the *PCSK9* gene with “gain of function—GOF”, hence promoting LDLR degradation [129]. Two years later, lower plasmatic levels of LDL-C were associated with the existence of two “loss of function mutations—LOF” in *PCSK9* that resulted in elevated LDLR concentration in hepatic cells [130,131]. All these findings pointed to PCSK9 protein inhibition as a new potential lipid-lowering therapeutic target. To reduce CVD, PSCK9 inhibition has been set as a therapy in patients with FH, in those resistant to statin treatment or in subjects that did not reach the LDL-C goal levels at maximum statin dose [132,133]. By contrary, no recommendations exist today for T2DM patients’ treatment.

### 4.1. Biology of PCSK9 in Lipid Metabolism and Vascular Homeostasis

PCSK9, also known as neural apoptosis-regulated convertase 1, is the ninth member of the secretory serine proteases of the subtilase family. Although it is mainly expressed by hepatocytes, PCSK9 is also present in the small intestine, kidney, pancreas, brain [128,134,135], and ischemic heart [136]. The 25-kb *PCSK9* human gene on chromosome 1p32 has 12 exons and 11 introns and is under the regulation of SREBP-2 (the sterol regulatory element-binding protein) [132,135]. It encodes a 692 amino acid serine protease that is synthesized as an inactive precursor, pre-proPCSK9. The pre-proPCSK9, stored in the ER, is processed to pro-PCSK9 and this last into mature PCSK9 which is bound to a cleaved prodomain that acts as a catalytic inhibitor and chaperone until its secretion into the circulation. The prodomain-mature PCSK9 heterodimer, with a plasma half-life of 5 min, binds to high-affinity specific proteins, leading to their intracellular degradation [135].

PCSK9 function is to regulate the amount of LDLR by forming a PCSK9-LDLR complex in the hepatocyte surface which is internalized by endocytosis and engaged into endosomal-lysosomal degradation [135,137]. PCSK9 can also bind to LDLR intracellularly through a Golgi-lysosome pathway for degradation [135,138].

Recent findings indicate the participation of PCSK9 in other processes beyond lipid homeostasis such as cell cycle, apoptosis, and inflammation with a potential effect on atherosclerosis [139]. Thus, LPS upregulate PCSK9 in liver and kidney in mice, in human EC and VSMCs [140]. Consistently, the injection of LPS into *Pcsk9* deficient mice resulted in less synthesis of pro-inflammatory IL-6 and TNFα cytokines and reduced expression of adhesion molecules in ECs and VSMCs [141]. 

*PCSK9* expression is promoted by TNF in liver and VSMCs and by oxidized LDL in ECs, macrophages, VSMCs, and DC [140,141]. In experimental atherosclerosis, PCSK9 is mostly found in VSMCs, although its expression is LDLR-dependent, as *Ldlr-/-* mice do not display *Pcsk9* expression [142]. Notably, *Pcsk9* expression is localized in the artery branches with low shear stress and mirrors cytokine expression of these sites indicating a proatherogenic effect [142]. On the other hand, *Apoe-/-* mice overexpressing human *PCSK9* specifically in the bone marrow displayed augmented levels of proinflammatory Ly6C^hi^ monocyte infiltration in atheromas. Moreover, human PCSK9-overexpresing macrophages displayed enhanced pro-inflammatory *Tnf* and *Il1b* genes and diminished anti-inflammatory *Il10* and *Arg* genes [143]. In agreement with these, in vivo Pcsk9 gene silencing by lentiviral transduction of Apoe-/- mice decreased lesion size and macrophage infiltration and expression of *Nfkb, Tnfa, Il1*, and *Tlr4* in atherosclerotic plaques [144]. In macrophages, PCSK9 stimulates proinflammatory cytokine expression and foam cell formation by upregulating *Sra* and *Cd36*. Other actions of PCSK9 include activation of T cells, monocyte migration, and VSMC apoptosis [142]. Altogether indicates a role of PCSK9 in atheroma lesion development by regulating inflammatory pathways. Notwithstanding, the clinical relevance of these anti-inflammatory actions remains to be established, as patients treated with PSCK9 inhibitors (PSCK9i) do not display altered levels of the hs-CRP inflammatory marker [142].

### 4.2. Rational for PCSK9 Inhibition as a Potential Therapy

The main rational for the development of PCSK9i as CVD therapy were the positive correlation between high PCSK9 levels and increased CVD risk and the association between different *PCSK9* polymorphisms and vascular illness [145,146]. Moreover, the moderately-high prevalence and the lack of harmful effects of *PCSK9* LOF mutations in healthy individuals suggested a high safety of a potential PCSK9 therapeutic inhibition [132,146].

On the other hand, experimental animal data also gave a foundation for the development of PCSK9i. *Pcsk9-/-* mice displayed up to 80% decrease in plasma LDL-C levels due to increased hepatic LDLR and LDL clearance [147], which resulted in dramatic reductions in aortic atherosclerosis [148].

To date, up to nine PCSK9 inhibitory strategies, acting either preventing its binding to the LDLR, or its maturation, secretion, or synthesis, have been or are being developed [149]. These therapies, described below and summarized in Table 4, include the use of monoclonal antibodies (mAb) against PCSK9 [150], antisense oligonucleotides (ASOs), small interfering RNA (siRNAs), vaccines, and small molecules [151].

#### 4.2.1. PCSK9 Monoclonal Antibodies

The only current clinical therapy to inhibit PCSK9 function in use is the administration of human mAb against soluble PCSK9. A single intravenous injection of the PCSK9 mAb, alirocumab, in hyperlipidemic APOE*3Leiden.CETP transgenic mice, decreased non-HDL-C, inflammatory parameters, and atherosclerosis lesion size and improved plaque stability in a dose-dependent manner [152]. Similarly, administration of a PCSK9 mAb reduced about 80% the levels of LDL-C in non-human primates [153]. An improved version of this mAb, with greater antigen affinity and resistance to degradation, achieved a reduction of around 40% in LDL-C levels at lower doses and an efficiency of 2.8 times longer [154]. 

The two monoclonal antibodies approved as a PCSK9-blocking therapies are alirocumab (SAR236553/REGN727 from Regeneron Pharmaceuticals/Sanofi) and evolocumab (AMG145 from Amgen). These are currently prescribed to reduce the risk of atherosclerotic CVD and lower LDL-C levels in patients with FH and resistance to statin therapy, among others [150,164]. Alirocumab is a human IgG1 mAb that strongly binds PCSK9 with a maximum effect between 8 and 15 days after its subcutaneous administration [165]. Evolocumab is a human mAb of the isotype G2 against PCSK9 with a maximum efficacy between the first and second week after subcutaneous administration [166]. 

Evolocumab was tested in the FOURIER (Further Cardiovascular Outcomes Research with PCSK9 Inhibition in Subjects with Elevated Risk [NCT01764633]) and OSLER (Open-label Extension Study of Evolocumab [NCT01439880]) trials [156,157], and alirocumab was evaluated in the ODYSSEY trial (Evaluation of Cardiovascular Outcomes After an Acute Coronary Syndrome With Alirocumab) [158,159]. These showed increased hepatic LDLR and reduced circulating levels of LDL-C by up to 60% as well as VLDL [167]. Both alirocumab and evolocumab slightly increased HDL levels and apolipoprotein A1 and diminished plasmatic lipoprotein(a) [132]. A current meta-analysis have shown that PCSK9 antibodies reduce nonfatal cardiovascular events and all-cause mortality [156,160], improve several atherogenic events [150], and promote plaque regression [168]. 

Notwithstanding, the use of mAbs as PCSK9 inhibitors for CVD therapy holds several limitations, such as the high cost (about 14,500 USD per patient each year), a limited lifetime, and administration frequency of twice a month [149]. Because of this, novel approaches to pharmacologically inhibit PCSK9 are under development consisting of gene silencing either with siRNAs or by using ASOs. 

#### 4.2.2. Small Interfering RNA (siRNA) 

The mechanism of action of *PCSK9* gene silencing uses a duplex 20 bp RNA molecule acting as a siRNA. Within the cell, it renders an anti-sense strand that binds to the mRNA gene, generating an incomplete duplex RNA later processed by DICER [151], thus preventing *PCSK9* translation and increasing LDLR protein hepatic content. 

Pre-clinical trials have shown that *PCSK9* gene silencing by siRNA reduced cholesterol levels in monkeys by 60% due to a 50–70% decrease in the *PCSK9* mRNA levels [155]. *PCSK9* siRNA suppressed the inflammatory response by diminishing the generation of ROS and LDL oxidation-induced apoptosis in ECs and macrophages, respectively [145]. In clinical trial phase I, this same *PCSK9* siRNA reduced LDL-C levels by 40% [161] but the effects were not lasting and an improved version of the siRNA was developed giving rise to Inclisiran (ALN-60212 from Alnylam) [149,169]. Inclisiran has been designed for hepatic cellular internalization, does not induce immunogenic reactions, and has a greater resistance to degradation by nucleases [169]. In phase I clinical trial, intravenous injection of Inclisiran, a siRNA in a lipid nanoparticle (ALN-60212), reduced the levels of PCSK9 and LDL-C by 74.5% and 50.6% respectively, with lasting effects between 3 to 6 months and decreases in non-HDL-C and apolipoprotein B (apoB) [170]. Phase II trial ORION-1, which evaluated different Inclisiran doses in patients who had a history of atherogenic CVD or high atherosclerotic CVD-risk with elevated LDL-C levels, showed a dose-dependent decrease in *PCSK9* and LDL-C levels [162]. Besides, patients who received two doses presented a reduction of 69.1% and 52.6% of PCSK9 and LDL-C [151,162], without adverse reactions [169]. Results of ORION phase III trials (ORION 10 and 11) have shown the effectivity of Inclisiran in patients with CVD, CVD risk or with elevated LDL-C levels despite statin-treatment at the maximum dose, with reductions in LDL-C, non-HDL-C, and apoB [163]. 

Because its potent and long-lasting effect lowering LDL-C levels, the administration frequency (once/twice per year) [149], low cost and easier long-term storage, Inclisiran holds great promise as an hypolipemiant agent.

### 4.3. Other Developing Approaches to PCSK9 Inhibition

Other drug strategies to inhibit PCSK9 function include the use of ASOs (antisense oligonucleotides), small-molecules inhibitors, PCSK9 vaccination or the CRISPR/Cas9 system (clustered regularly interspaced short palindromic repeat associated protein 9) to lower PCSK9 concentration [151]. These approaches, in preclinical phase or phase I [150], are expected to offer a number of several additional benefits such as prolonged effects, easier production and lower cost [149]. The administration of an ASO, which is a 15–30 pb single-strand DNA that hybridizes with *PCSK9* mRNA and ensues RNase H degradation route [164], resulted in a high specific decrease of LDL-C and PCSK9 levels of 25–50% and 50–85%, respectively, in a phase I clinical trial [151]. Notably, berberine, a natural product obtained from a plant, and its derivatives have been shown to inhibit the transcription of *PCSK9*, and therefore it could also be useful in the management of patients with CVD risk [149,168]. 

## 5. Conclusions

**Figure 2:**
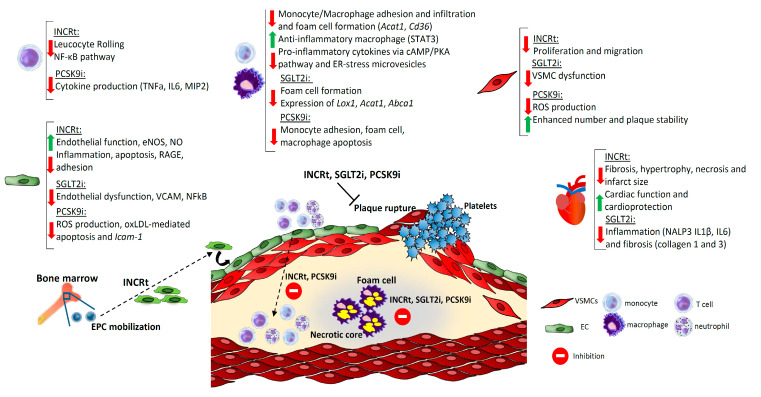
Common mechanism of action of incretin-therapies, SGLT2i and PCSK9i in immune and vascular cells involved in atherosclerosis progression and in cardiomyocyte function. The main protective mechanisms of PCSK9i and INCRt in T cells include the impairment of leukocyte rolling and a decrease in NFkB activation and cytokine secretion. Both PCSK9i and INCRt also reduce monocyte adhesion and recruitment while in macrophages all three class drugs decrease macrophage foam cell formation. INCRt also decreases inflammatory genes in macrophages and PCSK9i impairs restrains proatherogenic apoptosis. Endothelial function is improved by all three class drugs by several mechanisms such as enhanced eNOS, reduced adhesion of leukocytes and inflammatory molecules and diminished ROS production. Notably, INCrt also promote endothelial cell repair by the mobilization of EPC from the bone marrow. In VSMCs’ INCRt decrease proliferation and hyperplasia, PCSK9i promotes plaque stability by enhancing VSMC number, and SGLT2i reduce their dysfunction. All these mechanisms lead to a decrease in leukocyte retention in the subendothelial space, less foam cell formation, reduced necrotic core and prevention of plaque rupture that, in patients, ensues the acute cardiovascular events. Lastly, among the actions observed in cardiomyocytes stands out the improved cardiac function by INCRt and SGLT2i by decreasing inflammation and fibrosis and necrosis. EPC: endothelial progenitor cell; INCRt: incretin-therapies; SGLT2i: sodium-glucose co-transporter 2 inhibitor; PCSK9i: proprotein convertase subtilisin kexin 9 inhibitor.

The investigations reviewed here indicate that the new emergent clinical therapies designed to restore metabolic homeostasis have major effects on the cardiovascular system beyond metabolic control. In addition to their expected anti-diabetic and lipid-lowering actions, the main common mechanisms for CVD prevention and atheroprotection of the three class drugs in the studies reviewed here are a direct modulation of vascular and inflammatory cell phenotypes and an improvement of the vascular and cardiomyocyte function. These effects of the incretin-therapies, SGLT2i and PCSK9i in vascular, immune cells and cardiomyocytes have been summarized in Figure 2. These have been shown to prevent early stages of atherosclerosis such as decreased leukocyte adhesion, endothelial dysfunction, immune cell recruitment, and foam cell formation. Moreover, several animal studies also demonstrated that these strategies stabilize atheromas and diminish necrotic cores of advanced plaques. A promising future of incretin-based therapies and SGLT2i for their use in cardiovascular prevention, and possibly, the use of small molecules as PCSK9i for more affordable therapeutics, is anticipated. Notwithstanding, completion of the ongoing clinical trials and further insight about the mechanisms of action of these drugs is needed to reduce the burden of CVD in the future.

## Figures and Tables

**Table 1:** Preclinical studies of incretin-based therapies in animal models of atherosclerosis, vascular injury, and myocardial infarction.

Animal Model	Incretin Therapy	Mechanism of Action	Reported Effects	References
*Apoe-/-* mice	DPP4i Anagliptin	Suppressed VSMCs proliferation and macrophages inflammatory responses	Restrained atherosclerosis	[34]
	DPP4i Linagliptin+HFD	Anti-inflammatory phenotype of macrophages	Improved atherosclerosis	[35]
	GIP active forms	Decreased VSMCs proliferation, monocyte infiltration, foam cell formation and related genes (*Cd36*, *Acat1*), and NF-kB-mediated inflammation in macrophages	Stabilization of atherosclerotic plaque	[36]
	GIP and GLP1	Suppressed foam cell formation	Reduced atheroma plaque	[37]
	Liraglutide	Suppressed *Acat1* expression and foam cell formation	Decreased atherosclerosis	[38]
		Anti-inflammatory macrophage polarization	Reduced atherosclerosis	[39]
	Exenatide+CS	Reduced oxidative stress and inflammation	Reduced plaque	[40]
	Exendin-4	Reduced monocyte adhesion and pro-inflammatory cytokines via cAMP/PKA pathway	Decreased lesion size	[41]
APOE*3-Leiden.CETP mice	Exendin-4	Decreased monocyte recruitment and adhesion and foam cell formation	Reduced atherosclerotic lesions	[42]
*Apoe-/-Irs2+/-* mice	Liraglutide Lixisenatide	STAT3-mediated macrophage polarization to an anti-inflammatory phenotype	Decreased atherosclerosis, necrotic core	[43]
*Apoe-/-* and *Ldlr-/-* mice	Liraglutide Semaglutide+WD	Changes in inflammatory markers	Reduced lesion size	[44]
*Lldr-/-* mice	DPP4i	Decreased pro-inflammatory genes expression and macrophage content	Decreased plaque size	[45]
Arterial hypertension Angiotensin II-mouse model	Liraglutide	Reduced leukocyte rolling and neutrophils infiltration	—	[46]
C57Bl6 mice	Liraglutide+45% HFD	Reduced eNOS expression and ER-stress response	Reduced cardiac fibrosis, hypertrophy, and necrosis	[47]
Myocardial injury mouse model	Liraglutide	Enhanced GSK3β, PPARβ-δ, Nrf-2, and HO-1 genes	Reduced mortality, infarct size, and rupture	[48]
Ischemia-reperfusion injury rats	Lixisenatide	---	Reduced infarct-size, improved cardiac function	[49]
Restenosis mouse/rat model	Lixisenatide Exendin-4	Reduced VSMC proliferation	Neointimal hyperplasia	[50]
Diabetic rats	Liraglutide	Decreased macrophage ER-stress-induced secretion of microvesicles	Diminished atherosclerosis and intima thickening	[51]
	GLP1+adenovirus-mediated delivery	Reduced VSMC and monocyte migration and inflammation	Reduced intima thickening	[52]
Rabbits	DPP4i Anagliptin+CD	Reduced macrophage infiltration	Restrained atherosclerosis	[53]
WHHL rabbits	Lixisenatide	Reduced macrophage, calcium deposition, necrosis	Prevention of plaque growth and instability	[54]

ACAT1: acetyl-CoA acetyltransferase; CD: cholesterol diet; CS: chronic stress; eNOS: endothelial nitric oxide synthase; ER: endoplasmatic reticulum; HFD: high fat diet; VSMCs: vascular smooth muscle cells; WD: western diet; WHHL: Watanabe heritable hyperlipidemic.

**Table 2:** Clinical trials of incretin-based therapies.

Incretin-Therapy	Clinical Trial	Patients	Reported Effects	References
DPP4i saxagliptin	SAVOR-TIMI 53 [NCT01107866]	T2DM patients with CV risk	-Unaffected CV risk -Increased HF hospitalization rate	[29]
DPP4i alogliptin	EXAMINE [NCT00968708]	T2DM patients with ACS	-Unaffected CV death and hospital admission for HF	[30]
DPP4i sitagliptin	TECOS [NCT00790205]	T2DM patients with established CVD	-Unaffected MACE or hospitalization for HF risk	[60]
DPP4i Linagliptin	CAROLINA [NCT01243424]	T2DM patients and CV risk	-Unaffected CV risk	[61]
	CARMELINA [NCT01897532]	T2DM patients and CV risk and kidney disease	-Unaffected HF incidence -No influence of kidney disease -Reduced albuminuria	[31,62]
Lixisenatide (exendin-4 based)	ELIXA [NCT01147250]	T2DM patients with a recent ACS	-No effects on MACE, hospitalization for HF -Decreased SBP and heart rate	[63]
Exenatide (exendin-4 synthetic)	EXSCEL [NCT01144338]	T2DM patients with or without CVD	-Unaffected incidence of MACE, retinopathy or renal outcomes -Modest reduction in SBP but increased heart rate	[64]
			-Modest reduction in CV risk	[65]
Liraglutide (human GLP1A)	LEADER [NCT01179048]	T2DM patients at high CV risk	-Reduced rates of MACE and death -Reduced SBP and microvascular renal and retinal complications -Enhanced heart rate	[66]
			-Same benefits for polyvascular and single vascular disease	[67]
			-Reduced CV outcomes (MI/stroke) and CVD	[68]
			-Unaffected HF hospitalization and death risk after MI	[69]
			-Decreased rates of diabetic kidney disease	[70]
Semaglutide (human GLP1A)	SUSTAIN-6 [NCT01720446]	T2DM patients at high CV risk	-Reduced rates of CV death and non-fatal MI/stroke -Decreased SBP but enhanced mean pulse rate	[71]
	PIONEER 6 [NCT02692716]	T2DM patients with high CV risk	-Unaffected CV risk -Decreased SBP and LDL-C -Gastrointestinal adverse events	[72]
Albiglutide (modified human GLP1)	Harmony Outcomes [NCT02465515]	T2DM and CVD patients	-Decreased SBP but augmented heart rate -improved glomerular filtration rate -Reduced risk of MACE -Unaffected CV death	[73]
Dulaglutide (modified human GLP1)	REWIND [NCT01394952]	T2DM patients at high CV risk	-Unaffected all-cause mortality rate -Decreased SBP, pulse pressure and arterial pressure but enhanced heart rate -Reduced risk of CV outcomes, total CV, or fatal event burden	[74,75]
Dulaglutide (modified human GLP-1) and tirzepatide (LY3298176)	SURPASS-CVOT [NCT04255433]	T2DM patients with atherosclerotic CVD	Estimated completion date: October 2024	https://clinicaltrials.gov/NCT04255433
Efpeglenatide	AMPLITUDE-O [NCT03496298]	T2DM patients with high CDV risk	Estimated completion date: April 2021	https://clinicaltrials.gov/NCT03496298
NNC0090-2746 (RG7697) [NCT02205528]	Phase 2 trial	T2DM patients	-Improved glycemia control -Diminished body weight, cholesterol, and leptin.	[76]
Tirzepatide (LY3298176) [NCT03131687]	Phase 2 trial	T2DM patients	-Improved glycemic and body weight control -Enhanced pulse rate -Acceptable safety and tolerability profile	[77,78]

ACS: acute coronary syndrome; HF: heart failure; CV: cardiovascular; CVD: cardiovascular disease; DBP: Diastolic blood pressure; LDL-C: low density lipoprotein cholesterol; MACE: major adverse cardiovascular events; MI: myocardial infarction; SBP: systolic blood pressure; T2DM: type 2 diabetes mellitus.

**Table 3:** Summary of the studies about the effect of SGLT2i in preclinical animal models and in clinical trials of T2DM patients.

Drug	Animal Model	Effect on Lipids	Effect on Atherosclerosis	References
Dapagliflozin	KK/A^y^ mice	Decreased T-Chol, TG, and NEFAs (ipragliflozin and dapagliflozin)	-Decreased ED (CAMs and E-selectin) and plasmatic inflammatory parameters	[110]
Ipragliflozin				
Canagliflozin				
Luseogliflozin				
Empagliflozin				
Tofogliflozin				
Empagliflozin	*db/db* mice	No change in TG and T-Chol	-Reduced aortic and endothelial cell stiffness	[111]
Empagliflozin	ZDF rats	No change in TG, T-Chol, HDL, and LDL	-Reduced oxidative stress and inflammation -ED partially prevented	[112]
Dapagliflozin	*db/db* mice	---	-Lower arterial stiffness -Improved ED and VSMC dysfunction	[113]
Ipragliflozin Dapagliflozin	*STZ Apoe-/-* mice *db/db* mice	No changes in TG, HDL and T-chol	-Dapagliflozin decreased macrophage infiltration, atherosclerotic lesions and plaque size -Ipragliflozin decreased foam cell formation	[114]
Dapagliflozin	*Apoe-/-* mice	---	-Attenuated ED and VCAMs expression -Induced vasorelaxation	[115]
Empagliflozin	*Apoe-/-* mice	Decreased TG and increased HDL	-Decreased atherosclerotic plaque and inflammation	[116]
Canagliflozin	*Apoe-/-* mice	Decreased TG, T-chol and LDL	-Increased plaque stability -Reduced atherosclerosis and inflammatory parameters	[117]
Dapagliflozin	*ob/ob* mice	Decreased of TG	-Reduced expression of inflammatory parameters	[118]
Drug	Trial	Structural basis	-Effect on vascular and blood parameters, and MACE	References
Empagliflozin	EMPA-REG OUTCOME	C-glycosyl compound	-Decreased CV death (HR = 0.86), SBP and DBP -Increased HDL-C and LDL-C	[119]
Canagliflozin	CANVAS/CANVAS-R		-No effect (HR = 0.86) -Increased HDL-C and LDL-C	[120]
Canagliflozin	CREDENCE		-Decreased nonfatal stroke/MI and CV death (HR = 0.80)	[121]
Dapagliflozin	DECLARE-TIMI 58		-No effect (HR = 0.93) -Decreased SBP and DBP	[122]
Dapagliflozin	DEFINE-HF		-No decrease in HF (HR = 0.84)	[123]
Ertugliflozin	VERTIS-CV		-No effect (HR = 0.97) -Decreased SBP	[124]
Sotagliflozin	SCORED		-Decreased CV death (HR = 0.84)	[125]
Sotagliflozin	SOLOIST-WHF		-Decreased CV death (HR = 0.72)	[126]

CAMs: cellular adhesion molecules; CV: cardiovascular; CVD: cardiovascular disease; DBP: dyastolic blood pressure; ED: endothelial dysfunction; HF: heart failure; HR: hazard ratio for three-component; HDL cholesterol; LDL-C: LDL cholesterol; MACE; MACE: major adverse cardiovascular events; MI: myocardial infarction; SBP: systolic blood pressure; T-chol: total cholesterol; TG: triglycerides; VSM: vascular smooth muscle; VCAMs: vascular cell adhesion molecules.

**Table 4:** Pre-clinical and clinical trials studying PCSK9 inhibitors in the context of hypercholesterolemia.

Inhibition Strategy	Animal Models	Effects on Lipids	Effects on Atherosclerosis	References
PCSK9 mAb	Cynomolgus monkeys	Decreased LDL-C (80%), T-Chol (48%)	---	[153]
PCSK9 mAb	C57BL/6 mice Cynomolgus monkeys	Decreased LDL-C (40%)	---	[154]
PCSK9 mAb: alirocumab	APOE*3Leiden.CETP mice	Decreased non-HDL-C and TGs	-Decreased inflammation and atherosclerotic lesion -Increased plaque stability	[152]
siRNA PCSK9	Cynomolgus monkeys C57BL/6 mice Sprague–Dawley rats	Decrease of LDL-C and T-Chol (60%)	---	[155]
**Inhibition Strategy**	**Trial Name**	**Type of Patients**	**Reported Effects**	**References**
PCSK9 mAb: evolocumab	FOURIER/OSLER [NCT01764633; NCT01439880] Phase III	Patients with atherosclerotic CVD	-Increased LDLR and HDL-C -Decreased LDL-C (61%), Total-C (36.1%), TG (12.6%), and Lp(a) (25.5%). -Increased Apolipoprotein A1 and HDL-C -Reduced CV (12–19%)	[156,157]
PCAK9 mAb: alirocumab	ODYSSEY [NCT01507831] Phase III	ACS patients	-Increased hepatic LDLR and HDL-C (4%) -Decreased LDL-C (58%), Total-C (37.8%), TG (15.6%), and Lp(a) (29.3%) and MACE -Increased ApoA1 and HDL-C -Reduced CV events (48%)	[158,159]
PCSK9 mAb: alirocumab, evolocumab	Meta-analysis	Alirocumab or evolocumab-treated patients	-Decreased nonfatal CV events and mortality. -Improved atherogenic events	[150,160]
PCSK9 siRNA: ALN-PCS	Phase I dose-escalation study [NCT01437059]	Healthy adult volunteers	-Reduced LDL-C (40%)	[161]
PCSK9 siRNA: ALN-60212 (Inclisirian)	ORION 1 Phase II: [NCT02597127] Phase III 10, 11: [NCT03399370; NCT03400800]	Patients at CVD risk with elevated LDL-C and some receiving statins	-Reduced LDL-C (52.6%) relative to base-line, as well as apoB and non-HDL-C.	[162,163]

ACS: Acute coronary syndrome; ApoA1: Apolipoprotein A1; CV: cardiovascular; CVD: cardiovascular disease; HDL-C: HDL cholesterol; LDL-C: LDL cholesterol; LDLR: LDL receptor; Lp(a): lipoprotein (a); MACE: Major adverse cardiovascular events; Total-C: total cholesterol; TG: triglycerides.

## Data Availability

Data sharing not applicable.
